# Temperature and relative humidity differentially affect deltamethrin and malathion toxicity in the mosquito *Culex quinquefasciatus*

**DOI:** 10.7717/peerj.21565

**Published:** 2026-08-11

**Authors:** Donald Francis Ward, Joshua Kalmouni, Annika Avery, David Pino, Silvie Huijben, Krijn Petrus Paaijmans

**Affiliations:** 1 The Center for Evolution and Medicine, School of Life Sciences, Arizona State University, Tempe, AZ, United States of America; 2 Institute of Space Studies of Catalonia, Castelldefels, Spain; 3 Department of Physics, Universitat Politècnica de Catalunya.BarcelonaTech, Barcelona, Spain; 4 Simon A. Levin Mathematical, Computational and Modeling Sciences Center, Arizona State University, Tempe, United States of America; 5 WITS Research Institute for Malaria, Faculty of Health Sciences, University of Witwatersrand, Johannesburg, South Africa

**Keywords:** Culex quinquefasciatus, Insecticide toxicity, Deltamethrin, Malathion, Temperature coefficient, Relative humidity coefficient, Pyrethroid, Organophosphate, Phenotypic resistance, Vector control

## Abstract

**Background:**

Temperature and relative humidity are key drivers of ectotherm physiology, yet their combined effects on insecticide performance remain poorly understood. Here, we experimentally quantified how both temperature and relative humidity influence the toxicity of two widely used adulticides: the pyrethroid deltamethrin and the organophosphate malathion against *Culex quinquefasciatus* Say, 1823, a significant vector of West Nile virus.

**Methods:**

We exposed pyrethroid-susceptible females to deltamethrin or malathion using standard WHO tube bioassays conducted across two temperatures (20 °C, 27 °C) and three relative humidities (20%, 50%, 80%). Mortality at 24 hours post-exposure was analyzed using generalized linear models to quantify the independent effects of insecticide concentration, temperature, and relative humidity.

**Results:**

Deltamethrin toxicity increased at lower relative humidities (20% and 50% compared to the standard 80%) and, to a lesser extent, at a lower temperature (20 °C compared to 27 °C), yielding a pronounced negative relative humidity coefficient. In contrast, malathion toxicity showed a strong positive temperature coefficient but no relative humidity dependence.

**Discussion:**

Our findings provide what we believe is the first empirical evidence that temperature and relative humidity independently and differentially shape insecticide toxicity in mosquitoes. We hypothesize that these divergent responses are likely driven by distinct physicochemical and physiological mechanisms, with humidity-mediated effects on pyrethroid cuticular penetration and bioavailability contrasting with the temperature-driven metabolic activation of malathion. Our findings have important implications for interpreting phenotypic resistance assays, as field microclimates often diverge from standardized insectary conditions. This highlights the need to incorporate environmental context into resistance surveillance and vector control planning, which can, in turn, improve the predictive power of laboratory resistance assays.

## Introduction

Mosquito-borne diseases account for nearly 700 million cases and over one million deaths annually (Chilakam et al., 2023). These diseases include malaria, Zika, dengue, and West Nile virus. For many of these pathogens, effective pharmaceutical treatments or prophylactic measures are limited or unavailable. Consequently, mosquito control (including the use of chemical tools) remains the principal strategy for reducing human-mosquito contact (World Health Organization (WHO), 2017).

Common chemical interventions include the use of insecticide-treated bed nets and indoor residual spraying targeting *Anopheles* mosquitoes, the primary vectors of malaria (World Health Organization (WHO), 2024). Adulticide applications delivered *via* small aircraft, drones, trucks, or backpack sprayers are more commonly used to target *Aedes* (vectors of *e.g.*, Zika, dengue and chikungunya) and *Culex* mosquitoes (transmitting West Nile virus) (AMCA, 2021; AMCA, 2022). Furthermore, larvicides are often applied to aquatic breeding habitats to suppress immature mosquito stages (Okumu et al., 2025).

Unfortunately, mosquitoes have developed widespread resistance to many insecticides used in public health, undermining disease control and elimination efforts. Phenotypic resistance, defined as the observable ability of a mosquito to survive exposure to an insecticide, has been reported in multiple major vector species worldwide, including those transmitting malaria, Zika, dengue, and West Nile virus. The emergence of insecticide resistance is driven by intense selection pressure from repeated insecticide use, typically compounded by a limited rotation or combination of different chemical classes.

Currently, only a few insecticide classes are approved by the World Health Organization (WHO) for public health applications, with most being dependent on pyrethroids. Resistance to pyrethroids, carbamates, organochlorines, and organophosphates is widespread (Enayati et al., 2020; Moyes et al., 2017; Ranson et al., 2011; Richards et al., 2017), but resistance to newly introduced insecticides for vector control, such as chlothianidin, chlorfenapyr and brofanilide, is not yet prevalent (Minwuyelet et al., 2025). To extend the effective lifespan of existing and new insecticidal tools, it is critical to employ effective insecticide resistance management practices (World Health Organization (WHO), 2012). To do so effectively, surveillance of insecticide susceptibility across time and space is critical (Huijben & Paaijmans, 2018).

However, widely used phenotypic assays designed to monitor the emergence and spread of insecticide resistance (*i.e.,* technical resistance) do not reliably translate to the efficacy of vector control products in reducing mosquito populations under field conditions (*i.e.,* practical resistance) (Namias et al., 2021). Standard assays, such as the WHO tube test and the Centers for Disease Control and Prevention (CDC) bottle bioassay, enable the comparison of resistance profiles across time and geographic regions but fail to replicate the complex environmental and biological factors influencing insecticide performance. While these standardized assays allow environmental variables to be tightly controlled and can be adapted to explicitly test the effects of specific environmental conditions in laboratory settings, variables such as mosquito age, feeding or infection status, contact duration, and insecticide bioavailability all affect toxicity outcomes (reviewed in Namias et al., 2021).

Furthermore, both the CDC (Centers for Disease Control and Prevention (CDC), 2025b) and WHO (World Health Organization (WHO), 2022b) recommend that phenotypic resistance be evaluated in a climate-controlled insectary at 27 ± 2 °C and 75 ± 10% relative humidity (RH). These specific climate parameters are unlikely to reflect the environmental conditions in which many mosquito populations encounter insecticides in the field (Glunt, Blanford & Paaijmans, 2013).

**Figure 1:**
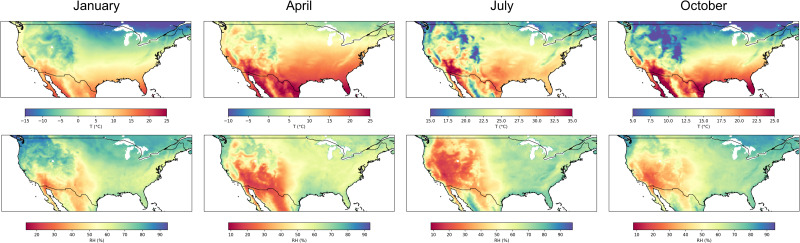
Seasonal variation in 2-m air temperature and relative humidity across the United States (2014–2023). Outdoor monthly mean 2-m temperature (top row) and relative humidity (bottom row) for January, April, July, and October across the United States during 2014–2023. Temperature and relative humidity fields are derived from the ERA5 reanalysis (Hersbach et al., 2020). Note that each panel uses its own color scale to highlight seasonal contrasts and to accommodate the large inter-monthly differences in temperature and relative humidity.

To illustrate this, we focus on the United States, where cases of West Nile virus (WNV) have been reported across the contiguous states. In 2025, 2,076 human cases were recorded in 47 states, approximately 1,434 of which were neuroinvasive, causing severe illness affecting the brain and spinal cord (Centers for Disease Control and Prevention (CDC), 2025a). WNV is primarily transmitted by *Culex quinquefasciatus* in the southern United States and by *Cx. pipiens* in the northern regions (Saarman et al., 2025). As shown in Fig. 1, the range of temperature and relative humidity conditions experienced by these mosquitoes in the continental U.S. varies substantially both between states at a given time and within states across seasons. Consequently, mosquitoes may encounter insecticides under climatic conditions that differ considerably from those used in standard laboratory assays.

Air temperature has a profound effect on insecticide toxicity because mosquitoes, as ectothermic organisms, rely on ambient temperature to regulate their body temperature and physiological processes (Angilletta, Niewiarowski & Navas, 2002; Huey & Kingsolver, 1989). Temperature variation influences insecticide toxicity primarily through two mechanisms (reviewed in Kalmouni et al., 2025). First, higher temperatures can accelerate the absorption and metabolic conversion of insecticides into their active toxic forms, leading to increased toxicity. Second, elevated temperatures can enhance the expression and activity of detoxifying enzymes, thereby increasing the rate of metabolic detoxification, which increases resistance. These opposing processes create a complex, and sometimes antagonistic, relationship between temperature and insecticide toxicity.

As such, temperature effects on insecticide toxicity are widespread but mechanistically complex and context dependent. To illustrate, and focusing on pyrethroids, deltamethrin exposure at temperatures both lower and higher than standard insectary conditions increased mortality (*i.e.,* showing a bimodal temperature-toxicity response) in both *Anopheles arabiensis*, and *An. funestus* (Glunt et al., 2018). In contrast, deltamethrin and permethrin exposure at temperatures higher than standard insectary conditions decreased mortality in *An. gambiae* mosquitoes (Agyekum et al., 2022). Similar negative temperature coefficients for pyrethroids have been reported in *Aedes aegypti*, where deltamethrin and permethrin exhibit reduced toxicity at elevated temperatures (Kalmouni et al., 2025; Whiten & Peterson, 2016). In *Cx. tarsalis*, deltamethrin was more toxic at warmer temperatures, but only at the lowest dose tested. At higher doses the impact of temperature on toxicity was negligible (Kalmouni et al., 2024). Note that the fact that the direction and magnitude of temperature effects are not always consistent across studies may partly reflect differences in experimental design or pseudoreplication in previous work (reviewed in Kalmouni et al., 2025).

In addition to temperature, water vapor pressure -and the related metric of relative humidity- is arguably one of the most important factors to consider when analyzing thermal responses in ectotherms (Anderson & Andrade, 2017; Guevara-Molina, Gomes & Camacho, 2020). Under low relative humidity conditions, ectotherms experience increased water loss, which can lead to dehydration and mortality. This effect is further exacerbated at high temperatures, where elevated evaporation rates intensify dehydration stress (How & Lee, 2014; Kleynhans & Terblanche, 2011; Li et al., 2019). Such physiological stress can, in turn, alter insecticide toxicity by affecting mosquito metabolism and/or cuticular permeability (Holmes & Benoit, 2019; Wang et al., 2011; see discussion).

The effect of relative humidity on insecticide toxicity has, to the best of our knowledge, not been established for mosquitoes. However, based on studies in other insects, an interaction between relative humidity and toxicity is likely. For example, etofenprox, a pyrethroid, was found to be less toxic at an RH of 55% than at an RH of 75% for larvae of the Mediterranean flour moth *Ephestia kuehniella*, for adults of the larger grain borer *Prostephanus truncatus*, and for larvae of the confused flour beetle *Tribolium confusum*, but not for *T. confusum* adults or adults of the lesser grain borer *Rhyzopertha dominica*, highlighting a potential life-stage effect (Boukouvala & Kavallieratos, 2022).

Rahman & Yadav (1987) examined several pyrethroids (deltamethrin, cypermethrin, permethrin, and fenvalerate) and found that toxicity against the cowpea weevil *Callosobruchus maculatus* and the pulse beetle *C. chinensis* increased with relative humidity when insecticides were applied in solution (*i.e.,* coated Petri dishes) but decreased when applied as dust formulations. Contrasting patterns have also been reported for other insecticide classes. For instance, the toxicity of DDT, an organochlorine, increased with higher RH in adults of the red flour beetle *Tribolium castaneum*, but decreased in larvae of the diamondback moth *Plutella maculipennis* (Pradhan, 1949). Similarly, the organophosphate pirimiphos-methyl exhibited greater efficacy against adults and larval instars of the mealworm beetle *Tenebrio molitor* at an RH of 55% compared to an RH of 75% (Kavallieratos et al., 2021), whereas Barson (1983) reported increased toxicity of another organophosphate, chlorpyrifos-methyl, with rising relative humidity in the sawtoothed grain beetle (*Oryzaephilus surinamensis)*. As such, the impact of relative humidity on insecticide toxicity in insects appears, as with temperature, to vary depending on the insecticide, formulation, insect species, and life stage.

Despite extensive research on the effects of temperature and relative humidity on insect physiology, the influence of relative humidity on mosquito susceptibility to insecticides—either alone or in combination with temperature—remains poorly understood. To date, no studies have directly examined how temperature and relative humidity interact to affect mosquito survival following insecticide exposure. Understanding these interactions is critical for improving the predictive power of laboratory bioassays and for optimizing vector control strategies under field-relevant conditions (Namias et al., 2021).

In this study, we investigate how variation in relative humidity and temperature influences the toxicity of the pyrethroid deltamethrin and the organophosphate malathion, against *Cx. quinquefasciatus*, a major vector of WNV in tropical and subtropical regions, particularly across the southern United States. These insecticides were selected to represent two major insecticide classes with distinct modes of action and widespread use in vector control, allowing for comparison of how environmental conditions influence toxicity across chemically and mechanistically different compounds. We hypothesize that increasing temperature will enhance malathion toxicity more strongly and consistently than deltamethrin toxicity in *Cx. quinquefasciatus*, with deltamethrin showing a more variable response. We assessed the effect of relative humidity without making a directional prediction.

## Materials & Methods

### Mosquito husbandry

Pyrethroid-susceptible *Cx. quinquefasciatus* mosquitoes (CMAVE strain, isolated from Gainesville, Florida, in 1995; obtained from the USDA-ARS, Center for Medical and Veterinary Entomology, Gainesville, Florida (Estep et al., 2024)) were reared in a climate-controlled chamber (Darwin Chambers Company, St. Louis, MO, USA) at 27 °C, 80% RH and a 12:12 h day:night cycle. Larval trays (∼500 larvae per tray) were refreshed once weekly with clean DI water and larvae were fed ground Cichlid fish food pellets (Aqueon, Franklin, WI, USA) every other day. Adult mosquitoes were provided with a 10% sucrose solution delivered through a cotton wool ball, which was replaced every other day. Parental mosquitoes were bloodfed using a Hemotek^®^ membrane feeding system and Type O+ human blood (Vitalant Research Institute, Denver, CO, USA). Egg rafts were collected in plastic 16 oz. deli containers 4-6 days after blood-feeding and added to 1 qt Sterilite plastic containers (up to 15 rafts per container), half-filled with DI water and 1/16 tsp of crushed alfalfa rabbit pellets (Oxbow, Omaha, NE, USA), for hatching and further larval development.

### Insecticide-treated papers

Insecticide-treated papers for WHO tube tests were prepared following standard procedures (World Health Organization (WHO), 2022a), using the gravimetric method which is based on accurate mass measurements rather than volume, to obtain precise concentrations (Jensen et al., 2022). In brief, selected insecticide concentrations (expressed as the percentage of active ingredient per unit volume of carrier oil) were prepared by mixing the required amount of insecticide in 2.0 ml of acetone, which was subsequently mixed with 0.7 ml olive oil (cat. No. 01514; Sigma-Aldrich). Filter paper (Whatman WHA1001929) was cut to 15 × 12 cm. Papers were coated with 2.7 mL of insecticide-oil mixtures in evenly dispersed droplets across the area of each paper. Papers were dried in a darkened fume hood for 24 h and subsequently wrapped in aluminum foil and stored at 4 °C until use (within three months), as per WHO guidelines (World Health Organization (WHO), 2022a).

Deltamethrin (Pestanal, cat. No. 45423; Sigma-Aldrich) concentrations were prepared at 0% (control, oil + acetone only), 0.0075%, and 0.015%, respectively 0.3× and 0.6 × the diagnostic dose for *Cx. quinquefasciatus* (World Health Organization (WHO), 2022b). For malathion (Pestanal, cat. No. 36143; Sigma-Aldrich), concentrations were prepared at 0% (control), 0.3125%, 0.625%, and 1%, or 0.06×, 0.13× and 0.2× the diagnostic dose (World Health Organization (WHO), 2022b). These values were based on initial pilot tests under standard insectary conditions that resulted in a range of mortality greater than 0% and smaller than 100%. Four papers were prepared for each concentration, which were randomly assigned to different treatment groups and replicates.

### Insecticide exposure

Mosquitoes were exposed to deltamethrin, malathion, or the control using the standard procedure for WHO tube bioassays (World Health Organization (WHO), 2022b). Exposures occurred at two temperatures (20 °C and 27 °C) and three relative humidities (20%, 50%, and 80%) in two reach-in environmental chambers (Caron Scientific, Model: 6025-1; Marietta, OH, USA). The selected humidity treatments span ecologically relevant conditions, ranging from relatively dry environments typical of some operational field settings to more humid conditions consistent with standard laboratory assays. This range was chosen to capture the variability in relative humidity that mosquitoes are likely to encounter in real-world scenarios, while still allowing for a direct comparison with established testing protocols.

All temperature and RH combinations were tested in random order, across three replicates. Each replicate was performed on a newly-reared batch of mosquitoes from a continuous lab colony to avoid pseudoreplication. All concentrations, including a 0% control, were evaluated simultaneously within a single assay (*i.e.,* one temperature-RH combination), and two assays (different temperature-RH combinations) were run concurrently. Temperature and relative humidity were monitored using dataloggers (SHT85-KHT, SensoScientific, CA, USA; HTP.xw, SensoPush, NY, USA) during all trials to assess the accuracy of environmental chambers (Fig. S1). Unfortunately, half of the data recordings from the deltamethrin trials, distributed randomly across all temperature-RH combinations, were lost prior to analysis due to a server communication issue. Because assays were conducted in randomized order and missing records were not concentrated within specific treatment groups, systematic bias is considered unlikely. For all experiments, environmental conditions were verified to be within the expected range based on observations taken before, during, and after exposure.

Before each exposure, approximately 25 two- to five-day-old female mosquitoes were aspirated into WHO holding tubes and acclimatized for 1 h, without access to sugar water, at their respective testing temperature and RH condition. Mosquitoes were then transferred to an exposure (or control) tube and exposed, at their experimental climate condition, for 1 h without access to sugar water, after which they were transferred into a small holding cage (BugDorm, 24.5 × 24.5 × 24.5 cm) and placed again at the experimental climate condition. Mosquitoes were kept in this holding cage for 24 h, at a photoperiod of 12:12 h (L:D) and provided with 10% sucrose solution *ad libitum*. Knockdown was assessed at 1-hour post-exposure and mortality at 24 h post-exposure. If mortality in the control group at 24 h post-exposure exceeded 20%, all concurrent tests for that replicate were discarded and repeated (World Health Organization (WHO), 2022b).

### Data analysis

Mortality 24 h post-exposure was corrected using Abbott’s formula when control mortality exceeded 5% (Abbott, 1925). Corrected mortality proportions were analyzed using generalized linear models (GLMs) with a logit link function and a quasibinomial distribution to account for overdispersion in the data (dispersion parameters were 2.21 and 5.47 in the final deltamethrin and malathion models, respectively). Observations were weighted by the number of mosquitoes assayed per replicate. Fixed effects included insecticide concentration (Deltamethrin: low/high; Malathion: low/intermediate/high), air temperature (20/27 °C), and relative humidity (20/50/80%). Three-way interaction terms were included initially; however, F-tests of the analysis of deviance indicated these were not significant (Table S1) and were therefore removed from the final model. The final model retained only the main effects of insecticide concentration, temperature, and relative humidity, which were assessed using analysis of variance (ANOVA)(F-tests). Separate models were run for the deltamethrin and malathion assays, odds ratios with corresponding 95% confidence intervals are reported in Tables S2 and S3 respectively. All analyses were conducted in R version 4.4.1 (R Core Team, 2025).

## Results

A total of 3,434 mosquitoes were included in the study, across 126 tubes in 36 unique assays. The average number of mosquitoes per tube was 27.3 (SD = 2.1; range 18–32). Mean control mortality was 1.1% (SD = 2.9%; range 0–14.2%), with Abbott’s correction used in three assays. The measured temperatures during the experiments with recorded data (see methods) were 20.2 °C (SD = 0.3; range 19.6–21.1) and 27.0 °C (SD = 0.3; range 26.5–27.7). The measured relative humidity were 19.8% (SD = 0.7; range 17.3–26.2), 47.6% (SD = 2.0; range 37.7–54.2), and 79.5% (SD = 3.5; range 62.1–87.4; Fig. S1).

Early knockdown responses following deltamethrin exposure were similar across treatments as temperature and relative humidity had no impact on knockdown 1-hour post-exposure (Fig. S2; Temperature: *F*_1,31_ = 2.1, *p* = 0.16; RH: *F*_2,31_ = 1.3, *p* = 0.29). However, the WHO tube test defines resistance status based on 24-hour post-exposure mortality. The deltamethrin concentration of 0.015% resulted in significantly greater 24-hour post-exposure mortality compared to 0.0075% (mortality: 87.0% *vs* 65.6%; *F*_1,31_ = 35, *p* < 0.001).

**Figure 2:**
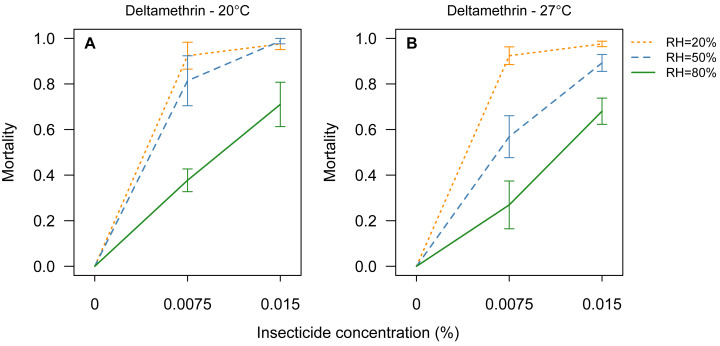
Mean 24-h post-exposure mortality of *Culex quinquefasciatus* exposed to deltamethrin at 20 ^∘^C (A) or 27 ^∘^C (B) across three relative humidities (20%, 50%, and 80%). Error bars represent standard error of the mean. Mean 24-hour post-exposure mortality for mosquitoes exposed to different doses of deltamethrin at 20 ^∘^C (A), or 27 ^∘^C (B) and RH = 20% (dotted orange line), 50% (dashed blue line), or 80% (solid green line). Error bars represent standard error of the mean of three independent replicates.

Mortality was influenced by both temperature and relative humidity during exposure. Mortality significantly decreased with increasing relative humidity (*F*_2,31_ = 44, *p* < 0.001; OR for 80% RH *vs* 20% RH = 0.04, 95% CI [0.02–0.10]). To illustrate, at the standard insectary air temperature of 27 °C and a deltamethrin concentration of 0.015%, mortality was 97.6% at RH = 20%, 89.2% at RH = 50%, and 68.0% at RH = 80%. This difference was even larger at the deltamethrin concentration of 0.0075% (92.4, 56.9, and 26.9% mortality, respectively). Additionally, a negative temperature coefficient was found, meaning increased mortality was observed at the lower temperature. However, this temperature effect was modest in comparison to the effect of relative humidity (*F*_1,31_ = 4.8, *p* = 0.036; OR for 27 °C *vs* 20 °C = 0.55, 95% CI [0.32–0.94]; Fig. 2, Table S2). No significant interactions between temperature and relative humidity were observed (Table S1).

The effects of temperature and relative humidity on malathion toxicity were distinct from those of deltamethrin. Early knockdown responses following malathion exposure were greatly increased at higher temperatures (*F*_1,48_ = 47, *p* < 0.001), with intermediate relative humidity causing slightly reduced knockdown (*F*_2,48_ = 5.1, *p* < 0.001; Fig. S3). For overall 24-hour post-exposure mortality, increased dosages resulted in increased mortality (*F*_2,48_ = 84, *p* < 0.001), with average mortalities of 21.8% (0.3125% malathion concentration), 54.3% (0.625% malathion concentration), and 93.5% (1% malathion concentration).

**Figure 3:**
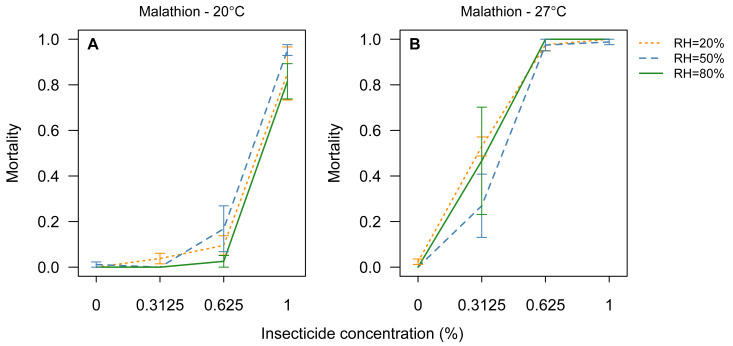
Mean 24-h post-exposure mortality of *Culex quinquefasciatus* exposed to malathion at 20 ^∘^C (A) or 27 ^∘^C (B) across three relative humidities (20%, 50%, and 80%). Error bars represent standard error of the mean. Mean 24-hour post-exposure mortality for mosquitoes exposed to different doses of malathion at 20 ^∘^C (A), or 27 ^∘^C (B) and RH = 20% (dotted orange line), 50% (dashed blue line), or 80% (solid green line). Error bars represent standard error of the mean of three independent replicates.

In contrast to deltamethrin, relative humidity did not have an impact on the toxicity of malathion (*F*_2,48_ = 0.28, *p* = 0.76). However, a very strong positive temperature coefficient was found, with higher temperatures resulting in higher mortality (*F*_1,48_ = 122, *p* < 0.001; OR for 27 °C *vs* 20 °C = 2,400, 95% CI [300–19,500]; Fig. 3, Table S3). No significant interactions were found between temperature and relative humidity (Table S1).

## Discussion

Our results reveal that environmental temperature and relative humidity differentially influence the toxicity of deltamethrin and malathion to *Cx. quinquefasciatus*, a major vector of WNV in the U.S. Deltamethrin toxicity increased significantly under conditions of reduced relative humidity and lower temperatures, with the effect of relative humidity being more pronounced than that of temperature. In contrast, malathion toxicity increased sharply with temperature but was unaffected by humidity. The temperature effect on malathion toxicity was apparent immediately after exposure, whereas the impact of temperature and relative humidity on deltamethrin manifested in the 24 h post-exposure.

These findings demonstrate not only that abiotic conditions can substantially modify insecticide efficacy, highlighting the importance of using standardized climatic conditions for bioassays, but also the need to contextualize such standardized technical resistance assays within field-relevant environmental conditions. However, these results should be interpreted within the relatively narrow thermal range tested (20–27 °C), which limits our ability to detect potential nonlinear temperature–toxicity relationships that may occur across broader environmental gradients (see limitations below).

Temperature is well recognized as a key determinant of insecticide performance in ectothermic organisms such as mosquitoes, affecting both toxicokinetics (*i.e.,* the movement of a substance through and metabolism within the body) and toxicodynamics (*i.e.,* a substance’s biochemical and physiological effects and the mechanisms by which it causes cellular and organismal damage) (Agyekum et al., 2022; Kalmouni et al., 2025; Mack & Attardo, 2024).

The positive temperature coefficient observed for malathion, an organophosphate, in our study is consistent with the hypothesis of temperature-enhanced bioactivation of this class. Malathion undergoes oxidative desulfuration to malaoxon, its active metabolite, catalyzed by cytochrome P450 monooxygenases (Jokanović, 2001). This enzymatic process may accelerate with increasing temperature, leading to higher internal concentrations of the active compound and thus higher mortality rates. Previous work on *An. gambiae* and *Cx. tarsalis* similarly reported increased organophosphate efficacy at elevated temperatures (Glunt et al., 2014; Kalmouni et al., 2024).

In contrast, deltamethrin exhibited a negative temperature coefficient, where lower temperatures yielded higher mortality. This pattern has been observed across several pyrethroid–insect combinations (*e.g.*, Scott & Georghiou, 1984; Whiten & Peterson, 2016), but the direction and magnitude of temperature effects are not always consistent across studies, which may partly reflect differences in experimental design or pseudoreplication in previous work (reviewed in Kalmouni et al., 2025).

Hypothetically, a negative temperature coefficient is expected, as pyrethroids act by delaying the closing of voltage-gated sodium channels in the insect nervous system (Narahashi, 1983). Because channel kinetics and neuronal excitability are temperature-dependent, higher temperatures can reduce binding affinity or shorten open-state duration, leading to diminished neurotoxic effects (Vijverberg et al., 1983). Moreover, detoxification enzyme systems, particularly mixed-function oxidases and esterases, show increased activity at higher temperature (Agyekum et al., 2022; Wang et al., 2021), potentially accelerating the metabolism of pyrethroids into less toxic derivatives. We hypothesize that these mechanisms together explain the observed negative temperature coefficient for deltamethrin.

The contrasting thermal responses of deltamethrin and malathion underscore how an insecticide mode of action determines the direction and magnitude of environmental effects. Pyrethroids, being lipophilic neurotoxins with low volatility, are often hypothesized to be more effective at lower temperatures where metabolic detoxification is slower and nerve recovery is prolonged. Organophosphates, in contrast, are thought to depend on enzymatic activation and thus tend to display positive temperature coefficients.

Compared to temperature, the influence of relative humidity on insecticide toxicity has received far less attention, particularly in mosquitoes. In our experiments, decreasing relative humidity from 80% to 20% substantially increased mortality for deltamethrin, while mortality resulting from malathion exposure remained unaffected. This strong relative humidity dependence for deltamethrin parallels observations in other insect taxa (Boukouvala & Kavallieratos, 2022; Rahman & Yadav, 1987).

However, as with temperature effects, humidity-toxicity relationships are not universally consistent across the literature. The same studies report the opposite pattern, with certain pyrethroids or other insecticide classes exhibiting greater toxicity at higher humidity, depending on the species and life stage (Boukouvala & Kavallieratos, 2022), and formulation used (Rahman & Yadav, 1987). These contrasting findings suggest that relative humidity may interact with species-specific physiology, cuticular properties, and insecticide formulation to produce a range of possible outcomes.

Nevertheless, for lipophilic pyrethroids such as deltamethrin, a negative humidity-toxicity relationship is mechanistically plausible, as lower relative humidity increases desiccation stress and cuticular permeability and reduces the likelihood of insecticide hydrolysis associated with high RH. From a physiological perspective, low relative humidity increases evaporative water loss and imposes desiccation stress (Chown, Sorensen & Terblanche, 2011), which can heighten susceptibility to insecticides. However, this explanation would predict a generalized response of increased susceptibility to insecticides, regardless of class. Because we found a strong relative humidity effect for a pyrethroid and no effect at all with an organophosphate, this explanation is less likely.

Another possible physiological hypothesis, which we did not directly test, involves the effect of relative humidity on cuticle composition. Dehydration may alter cuticular barrier properties, including the composition and organization of cuticular lipids, which increases permeability to hydrophobic compounds such as deltamethrin (Gibbs & Rajpurohit, 2010; Hadley, 1994). Conversely, at high humidity, increased cuticle hydration may modify the organization of cuticular lipids, potentially reducing the diffusion of lipophilic insecticides such as deltamethrin (Lockey, 1988). In contrast, malathion is hydrophilic (Tomlin, 2000); therefore, a lower water content in the cuticle would likely have little effect on its penetration or could even hinder it.

Alternatively, the observed results may arise from the chemical and physical behavior of the insecticide itself under different relative humidity conditions. Because pyrethroids are highly lipophilic, their distribution and persistence on treated surfaces may depend strongly on surface moisture. Under humid conditions, thin aqueous films may readily form on hydrophilic substrates (Santos & Verdaguer, 2016), providing a micro-aqueous environment that promotes partial hydrolysis of pyrethroids and reduces the amount of active compound available for mosquito uptake (Aiello et al., 2021; Laskowski, 2002). In contrast, malathion, being more hydrophilic and volatile, spreads more uniformly across filter paper and can evaporate readily regardless of ambient humidity. Indeed, earlier work showed that some organophosphate vapors, such as naled, exhibit greater volatilization at high relative humidity when deposited on hydrophilic, adsorptive substrates, whereas this effect diminishes or disappears on less sorptive surfaces (Ebeling & Wagner, 1965). Thus, the interplay between relative humidity and insecticide chemical properties, particularly hydrophilicity, lipophilicity, and volatility, may strongly influence the effective dose a mosquito receives, independent of physiological responses.

Finally, we hypothesize that behavioral changes under different relative humidity regimes may also explain our results. Mosquito activity generally increases with relative humidity (Brown et al., 2023). Under drier conditions, insects often reduce movement to conserve water, adopting resting postures that increase the duration of contact with treated surfaces, although opposite patterns have also been reported (Hagan et al., 2018). Such prolonged contact could increase the pickup and penetration of contact insecticides, especially when desiccation simultaneously compromises cuticular barrier function. Such behavioral modulation could contribute to the steeper mortality gradients observed across relative humidity treatments for deltamethrin, whose efficacy depends more on direct surface contact, whereas the more volatile malathion is likely less affected by differences in contact frequency. Whether such behavioral impacts also impact mortality in the WHO bioassay is unknown and data on activity patterns were not collected in our study.

The observed effects of temperature and relative humidity have important implications for how phenotypic resistance assays are interpreted and how operational control programs evaluate insecticide performance. Standardized WHO tube tests (World Health Organization (WHO), 2022b) and CDC bottle bioassays (Centers for Disease Control and Prevention (CDC), 2025b) are performed under fixed laboratory conditions (27 ± 2 °C and 75 ± 10% RH), which represent only a narrow slice of the environmental conditions under which mosquitoes encounter insecticides in the field (*e.g.*, Glunt, Blanford & Paaijmans, 2013; Fig. 1). For example, *Cx. quinquefasciatus* populations in the Pacific Southwest frequently experience evening temperatures of 20–35 °C with relative humidity often below 30% during the summer insecticidal fogging season (National Oceanic and Atmospheric Administration (NOAA), 2025; Fig. 1).

Our results indicate that pyrethroid toxicity increases substantially under such arid conditions. Consequently, a mosquito population that appears resistant when tested at 75% RH in the insectary may, in fact, be considerably more susceptible under the lower relative humidity regimes typical of operational environments in the Pacific Southwest. In other words, technical resistance measured under standardized laboratory conditions may overestimate true field resistance for pyrethroids, potentially leading to the underutilization of effective products. Conversely, organophosphate efficacy was unaffected by relative humidity but increased sharply with temperature. This suggests that field-relevant temperature variation, rather than humidity, will be the primary environmental modifier of organophosphate performance, with hotter field conditions amplifying malathion toxicity relative to laboratory assays. However, these implications should be interpreted with caution, as our experiments were conducted using residual-contact WHO tube assays under constant environmental conditions. Field applications, such as ultra-low-volume aerosol spraying, involve different exposure pathways and environmental dynamics that may alter insecticide performance.

These context-dependent differences echo the distinction between “technical resistance” (susceptibility measured under controlled, standardized conditions) and “practical resistance” (the reduction in field efficacy under real-world environmental conditions; Namias et al., 2021). Integrating environmental variability into resistance monitoring protocols, either by interpreting data through an environmental lens, or directly incorporating local microclimate into operational assessments, could improve predictions of field efficacy and support more locally appropriate insecticide selection.

More broadly, these findings reinforce the rationale for adhering strictly to standardized assay conditions when the goal is to compare technical resistance levels across space or time. If laboratories differ in temperature or humidity, susceptibility estimates may not be comparable. At the same time, programs should recognize that these standardized values do not necessarily reflect the conditions under which control products are deployed, and resistance data should be interpreted alongside the climatic context of field applications.

This study has several limitations. First, we used a fully susceptible laboratory strain of *Cx. quinquefasciatus*, and insecticide concentrations were selected to span sub-lethal to near-lethal ranges. In addition to known resistance mechanisms, different field populations of the same species may exhibit substantial variation in insecticide susceptibility due to underlying genetic, physiological, and ecological differences. Such population-level variation could also contribute to inconsistencies observed across studies, alongside the methodological differences highlighted above. As a result, the patterns reported here may not fully capture the range of responses present in natural populations. Field populations often harbor target-site mutations or metabolic resistance mechanisms that may interact with temperature or relative humidity in ways not captured here. For example, *kdr* alleles or elevated P450 expression could alter the relative contributions of penetration, detoxification, and activation under different environmental conditions (Kalmouni et al., 2025; Scott & Georghiou, 1984). Future work should, therefore, extend these experiments across multiple populations to assess the generality of these environmental effects under diverse genetic and ecological contexts.

Second, mortality was assessed at 24 h post-exposure. Although this aligns with WHO protocols, it does not capture delayed mortality, knockdown recovery, behavioral avoidance, or sublethal impacts on fecundity or host-seeking, all of which may be modulated by environmental stressors (Andreazza, Oliveira & Martins, 2021; Hubbard & Murillo, 2024; Viana et al., 2016).

Third, our experiments were conducted across only two discrete temperature treatments (20 °C and 27 °C). While these reflect commonly used laboratory and field-relevant conditions, this limited range constrains our ability to detect potential nonlinear temperature-toxicity relationships that may emerge across broader thermal gradients (Glunt et al., 2018; Whiten & Peterson, 2016).

Fourth, our experiments were conducted under stable climatic conditions, whereas natural environments fluctuate on hourly and daily timescales. Temperature-relative humidity interactions are often non-linear, and short-term fluctuations may produce effects that differ from those observed under constant conditions (Colinet et al., 2015; Lambrechts et al., 2011; Paaijmans et al., 2010). Incorporating diurnally fluctuating regimes would, thus, enhance ecological realism.

Fifth, because WHO and CDC assays rely on treated surfaces, humidity-driven processes such as surface hydrolysis and substrate porosity may influence toxicity outcomes in ways that are not applicable to airborne exposure in the field. As such, relative humidity coefficients derived from residual assays may not generalize to practical resistance under open field conditions. Field or controlled open-air trials are needed to validate whether these patterns hold operationally.

Finally, expanding this research to other chemical classes, such as carbamates, neonicotinoids, and newer pyrazoles, will help assess whether relative humidity dependence is unique to pyrethroids or general across all lipophilic insecticides.

## Conclusions

Our study provides, to our best knowledge, the first experimental evidence that temperature and relative humidity independently and differentially influence the toxicity of two major insecticide classes in mosquitoes. Malathion exhibited a strong positive temperature coefficient, consistent with enhanced metabolic activation at higher temperatures. In contrast, deltamethrin displayed a pronounced negative relative humidity coefficient, potentially reflecting reduced cuticular penetration or decreased residue availability under moist conditions.

These results demonstrate that insecticide efficacy is not fixed, but varies substantially with ambient environmental conditions. While standardized bioassays remain essential for assessing technical resistance and enabling comparisons across space and time, interpreting these outcomes through the lens of local microclimates is critical for understanding practical resistance and predicting field performance.

Incorporating environmental parameters, such as temperature and relative humidity, into resistance surveillance and operational planning will enhance the realism and predictive value of current bioassay protocols. As climate change continues to shift local thermal and relative humidity regimes, understanding how these abiotic factors modulate insecticide toxicity, and how these effects differ across chemical classes, will be increasingly important for maintaining effective vector control and prolonging the useful lifespan of insecticide-based interventions.

## Supplemental Information

10.7717/peerj.21565/supp-1 Supplemental Information 1 Measured temperature and relative humidity across experimental conditions during the acclimatization and exposure period Measured temperature (left columns) and relative humidity (right columns) in each temperature and relative humidity combination (rows) per experimental run (individual lines) per 15-minute intervals from 1-h acclimatization period to 24-hour mortality assessment.

10.7717/peerj.21565/supp-2 Supplemental Information 2 Mean 1-h post-exposure knockdown of *Culex quinquefasciatus* exposed to deltamethrin at 20 °C (A) or 27 °C (B) across three relative humidities (20%, 50%, and 80%). Error bars represent standard error of the mean Mean 1-hour post-exposure knockdown for mosquitoes exposed to different doses of deltamethrin at 20 °C (A), or 27 °C (B) and a relative humidity of 20% (dotted orange line), 50% (dashed blue line), or 80% (solid green line). Error bars represent standard error of the mean of three independent replicates.

10.7717/peerj.21565/supp-3 Supplemental Information 3 Mean 1-h post-exposure knockdown of * Culex quinquefasciatus* exposed to malathion at 20 °C (A) or 27 °C (B) across three relative humidities (20%, 50%, and 80%). Error bars represent standard error of the mean Mean 1-hour post-exposure knockdown for mosquitoes exposed to different doses of malathion at 20 °C (A), or 27°C (B) and a relative humidity of 20% (dotted orange line), 50% (dashed blue line), or 80% (solid green line). Error bars represent standard error of the mean of three independent replicates.

10.7717/peerj.21565/supp-4 Supplemental Information 4 F statistics, degrees of freedom (df), and p-values from analysis of deviance tests of three way and two-way interaction terms in quasibinomial logistic regression models of mosquito mortality for deltamethrin (left) and malathion (right)

10.7717/peerj.21565/supp-5 Supplemental Information 5 Odds ratios with 95% confidence intervals from the quasibinomial logistic regression model of mosquito mortality as a function of deltamethrin concentration, temperature, and relative humidity Odds ratios are reported relative to the reference conditions (0.0075% deltamethrin, 20 °C, and 20% RH)

10.7717/peerj.21565/supp-6 Supplemental Information 6 Odds ratios with 95% confidence intervals from the quasibinomial logistic regression model of mosquito mortality as a function of malathion concentration, temperature, and relative humidity Odds ratios are reported relative to the reference conditions (0.3125% malathion, 20 °C, and 20% RH).

10.7717/peerj.21565/supp-7 Supplemental Information 7 Mortality data for *Culex quinquefasciatus* exposed to deltamethrin and malathion across a range of doses, temperatures, and relative humidity conditions

## Additional Information and Declarations

### Data Availability

The following information was supplied regarding data availability: The data is available in the Supplemental Files.
