# Supplementary material for: Temperature and relative humidity differentially affect deltamethrin and malathion toxicity in the mosquito Culex quinquefasciatus
Source: PeerJ. 2026 Aug 11;14:e21565. doi: 10.7717/peerj.21565 (PMC13475386; doi:10.7717/peerj.21565)
Supplement: Supplementary file 1 [file peerj-14-21565-s001.pdf]

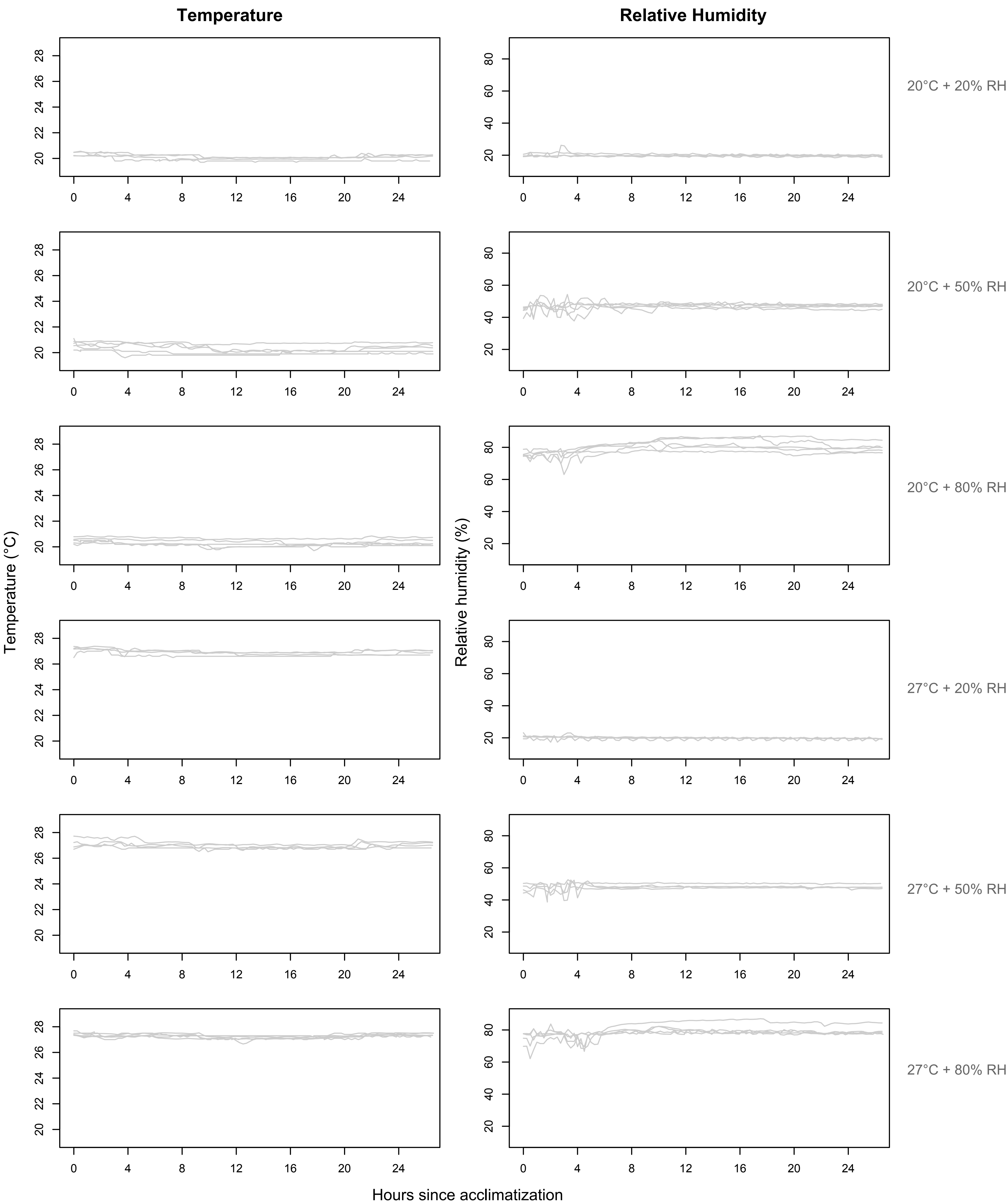
Temperature
Relative Humidity
20°C + 20% RH
20°C + 50% RH
20°C + 80% RH
27°C + 20% RH
27°C + 50% RH
27°C + 80% RH
Temperature (°C)
Relative humidity (%)
Hours since acclimatization
