# Supplementary material for: Temperature and relative humidity differentially affect deltamethrin and malathion toxicity in the mosquito Culex quinquefasciatus
Source: PeerJ. 2026 Aug 11;14:e21565. doi: 10.7717/peerj.21565 (PMC13475386; doi:10.7717/peerj.21565)
Supplement: Supplementary file 2 [file peerj-14-21565-s002.pdf]

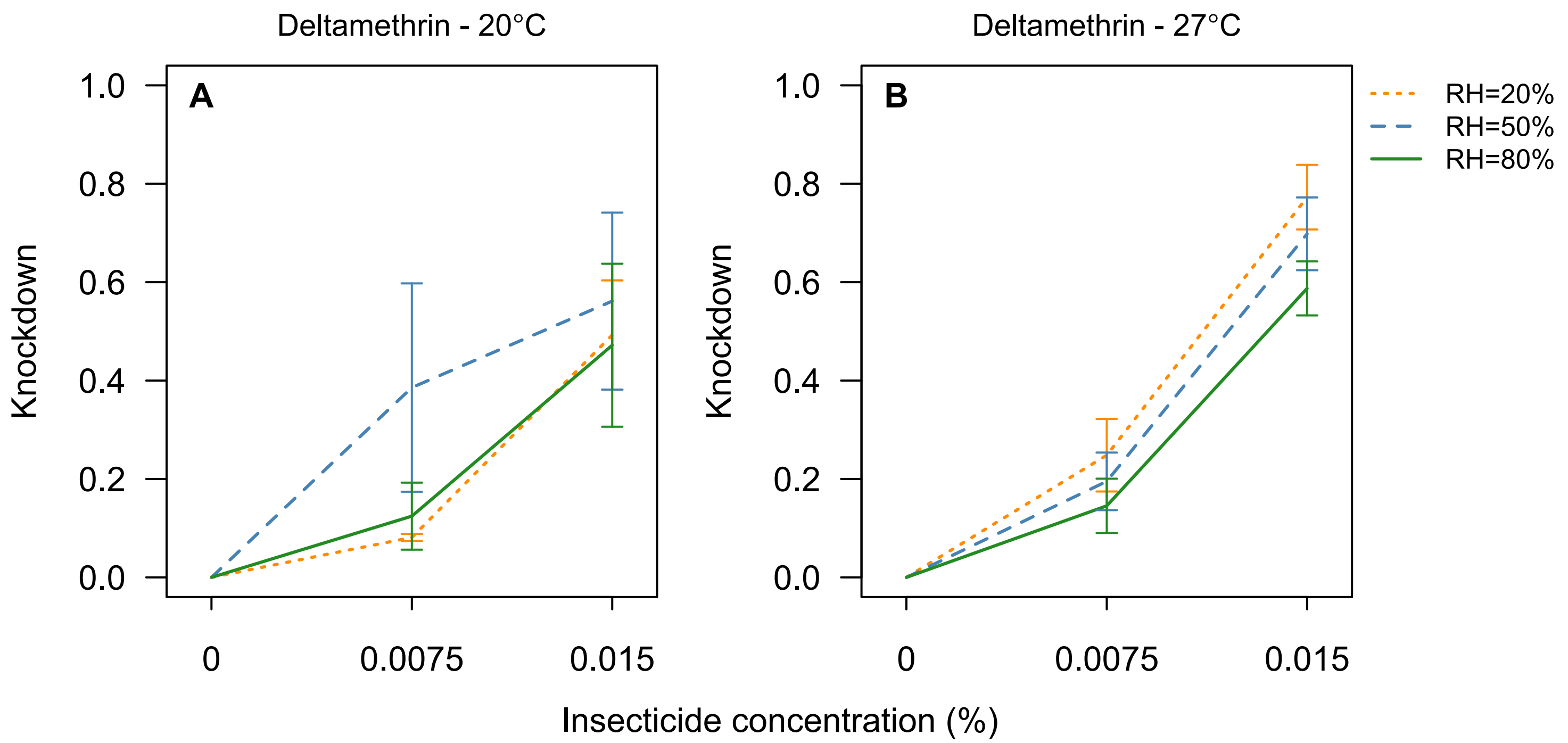

Deltamethrin - 20°C
Deltamethrin - 27°C
A
B
1.0
0.8
0.6
0.4
0.2
0.0
Knockdown
0
0.0075
0.015
RH=20%
RH=50%
RH=80%
Insecticide concentration (%)
