# Supplementary material for: Temperature and relative humidity differentially affect deltamethrin and malathion toxicity in the mosquito Culex quinquefasciatus
Source: PeerJ. 2026 Aug 11;14:e21565. doi: 10.7717/peerj.21565 (PMC13475386; doi:10.7717/peerj.21565)
Supplement: Supplementary file 4 [file peerj-14-21565-s004.docx]

**Table S1**. F statistics, degrees of freedom (df), and p-values from analysis of deviance tests of three way and two-way interaction terms in quasibinomial logistic regression models of mosquito mortality for deltamethrin (left) and malathion (right).

|  | Deltamethrin | | | Malathion | | |
| --- | --- | --- | --- | --- | --- | --- |
| Term | F-statistic | df | p-value | F-statistic | df | p-value |
| Concentration x Temperature x RH | 0.37 | 2 | 0.70 | 0.32 | 4 | 0.87 |
| Concentration x Temperature | <0.001 | 1 | 0.99 | 1.01 | 2 | 0.37 |
| Concentration x RH | 0.25 | 2 | 0.78 | 0.74 | 4 | 0.57 |
| Temperature x RH | 1.25 | 2 | 0.30 | 1.81 | 2 | 0.18 |
