# Supplementary material for: Temperature and relative humidity differentially affect deltamethrin and malathion toxicity in the mosquito Culex quinquefasciatus
Source: PeerJ. 2026 Aug 11;14:e21565. doi: 10.7717/peerj.21565 (PMC13475386; doi:10.7717/peerj.21565)
Supplement: Supplementary file 5 [file peerj-14-21565-s005.docx]

**Table S2**. Odds ratios with 95% confidence intervals from the quasibinomial logistic regression model of mosquito mortality as a function of deltamethrin concentration, temperature, and relative humidity. Odds ratios are reported relative to the reference conditions (0.0075% deltamethrin, 20°C, and 20% RH)

| Term | Odds ratio | 95% CI | p-value |
| --- | --- | --- | --- |
| Intercept | 15 | 6.7-36 | <0.001 |
| Concentration 0.015 (vs ref) | 5.2 | 2.9-9.3 | <0.001 |
| Temperature 27 (vs ref) | 0.55 | 0.32-0.94 | 0.037 |
| RH 50 (vs ref) | 0.22 | 0.09-0.54 | 0.002 |
| RH 80 (vs ref) | 0.04 | 0.02-0.10 | <0.001 |
