# Supplementary material for: Temperature and relative humidity differentially affect deltamethrin and malathion toxicity in the mosquito Culex quinquefasciatus
Source: PeerJ. 2026 Aug 11;14:e21565. doi: 10.7717/peerj.21565 (PMC13475386; doi:10.7717/peerj.21565)
Supplement: Supplementary file 6 [file peerj-14-21565-s006.docx]

**Table S3.** Odds ratios with 95% confidence intervals from the quasibinomial logistic regression model of mosquito mortality as a function of malathion concentration, temperature, and relative humidity. Odds ratios are reported relative to the reference conditions (0.3125% malathion, 20°C, and 20% RH)

| Term | Odds ratio | 95% CI | p-value |
| --- | --- | --- | --- |
| Intercept | 0.003 | <0.001-0.024 | <0.001 |
| Concentration 0.625 (vs ref) | 45 | 8.0-250 | <0.001 |
| Concentration 1.0 (vs ref) | 2400 | 300-19500 | <0.001 |
| Temperature 27 (vs ref) | 270 | 44-1700 | <0.001 |
| RH 50 (vs ref) | 0.77 | 0.28-2.1 | 0.62 |
| RH 80 (vs ref) | 0.69 | 0.25-1.9 | 0.47 |
